# A Systematic Review of Current Practices, Challenges, and Future Directions for the Use of Robotic Surgery in Otolaryngology in Greece

**DOI:** 10.7759/cureus.74458

**Published:** 2024-11-25

**Authors:** Maria Athina Tsitsika, Spyros Katsinis, Christos Damaskos, Stylianos Kykalos, Gerasimos Tsourouflis, Nikolaos Garmpis, Dimitrios Dimitroulis

**Affiliations:** 1 Department of Otolaryngology, Laiko General Hospital, Athens, GRC; 2 Hellenic Minimally Invasive and Robotic Surgery (MIRS) Study Group, Medical School, National and Kapodistrian University of Athens, Athens, GRC; 3 Department of Emergency Surgery, Laiko General Hospital, Athens, GRC; 4 N.S. Christeas Laboratory of Experimental Surgery and Surgical Research, Medical School, National and Kapodistrian University of Athens, Athens, GRC; 5 Second Department of Propedeutic Surgery, Laiko General Hospital, Medical School, National and Kapodistrian University of Athens, Athens, GRC; 6 Hellenic Minimally Invasive and Robotic Surgery (MIRS) Study Group, Laiko General Hospital, Medical School, National and Kapodistrian University of Athens, Athens, GRC; 7 Second Department of Propedeutic Surgery, Laiko General Hospital, Athens, GRC; 8 Department of Surgery, Sotiria General Hospital, Athens, GRC

**Keywords:** greece, obstructive sleep apnoea, otolaryngology (ent), robotic surgery, thyroidectomy, transoral robotic surgery (tors)

## Abstract

Robotic surgery is increasingly used in otolaryngology (ENT), particularly for complex head and neck procedures. It offers various advantages, including limited postoperative pain, excellent aesthetic results, better visualization in the surgical field, enhanced dexterity due to movement adjustment by the robotic system, and minimal complications and hospital stay. However, robotic systems' higher cost and limited availability are a burden for clinical applications. This systematic review is a detailed assessment that looks at the existing situation, problems, and prospects for robotic ENT surgery in Greece. It is based on the Preferred Reporting Items for Systematic Reviews and Meta-Analyses (PRISMA) criteria. The included studies were chosen based on specific criteria after a thorough inspection of electronic databases of clinical trials and medical journals (PubMed, Scopus, Web of Science). Despite steady adoption, Greece needs to catch up with other European countries in deploying robotic surgery technology. Various possible reasons may cause the small number of ENT robotic-assisted surgeries, including the high cost and the availability of robotic systems, mainly in large private or public hospitals in the main cities of Greece (Athens and Thessaloniki). Training on robotic systems is also very limited for surgery residents and young surgeons, while the learning curve of robotic-assisted surgeries in ENT is big. Peer-reviewed literature was analyzed to compare it with other European nations and investigate the economic, training, and geographic aspects that may be a burden for the rise of robotic surgery in Greece. Through the review scope, this study also provided recommendations concerning the implementation of robotic surgery in daily practice among surgeons in Greece and the difficulties that may arise regarding robotic surgery training in resource-limited countries.

## Introduction and background

Robotic-assisted surgery has altered surgical techniques in a variety of medical specializations, including otolaryngology (ENT) and head and neck surgery. The advantages of the robot-assisted surgery procedures are numerous: enhanced visualization, with 3D visualization and magnification of the operative field, elimination of the physiologic tremors, scale motion, use of multiarticulate instruments, reduction of fatigue during surgery, as the surgeon is sitting in an ergonomic position, as well as telesurgery availability [1,2]. However, robotic surgery in the ENT field does not come without disadvantages. The absence of haptic and tactile sensation is essential to a surgeon, considering the significance of the tension or the tissue resistance when tying a knot. Additionally, the robotic systems' size, weight, and cost require resources and space that may be limited [1].

The advantages of robotic systems, such as the Da Vinci, in precision, visualization, and dexterity make this technology particularly useful for complex head and neck operations [2,3]. Although there are various advantages to using robotic-assisted methods for surgeries in the ENT field, there are also some problems that arise, including the high cost of the surgery, extended operation duration, time needed for the setup of the robotic system, as well as limited availability due to high cost of obtaining such a robotic system. Thus, in developing or limited-resource countries, public hospitals may need more financial resources to obtain a robotic system, such as the Da Vinci, or pay for the surgeons to get adequate training for its safe use. For all the abovementioned reasons, Greece's adoption of robotic surgery in ENT has been sluggish due to financial constraints. Other variables that contribute significantly to this include limited access to new and advanced technology and differences in training and competence.

This systematic review provides a complete examination of the current state of robotic surgery in ENT in Greece, focusing on specific surgical procedures and outcomes. It also compares the situation in Greece with other European countries. Furthermore, this review aims to present the obstacles and future potential of robotic surgery in the Greek healthcare system.

## Review

Materials and methods

This systematic review was performed based on a predefined protocol and search strategy so that the authors would minimize bias. Based on this search, only four studies were performed in Greece and complied with all the inclusion and exclusion criteria [4-8]. This results in very little data in terms of ENT robotic surgery in Greece and reveals a gap in the literature that could initiate high-quality retrospective or prospective clinical studies.

**Figure 1:**
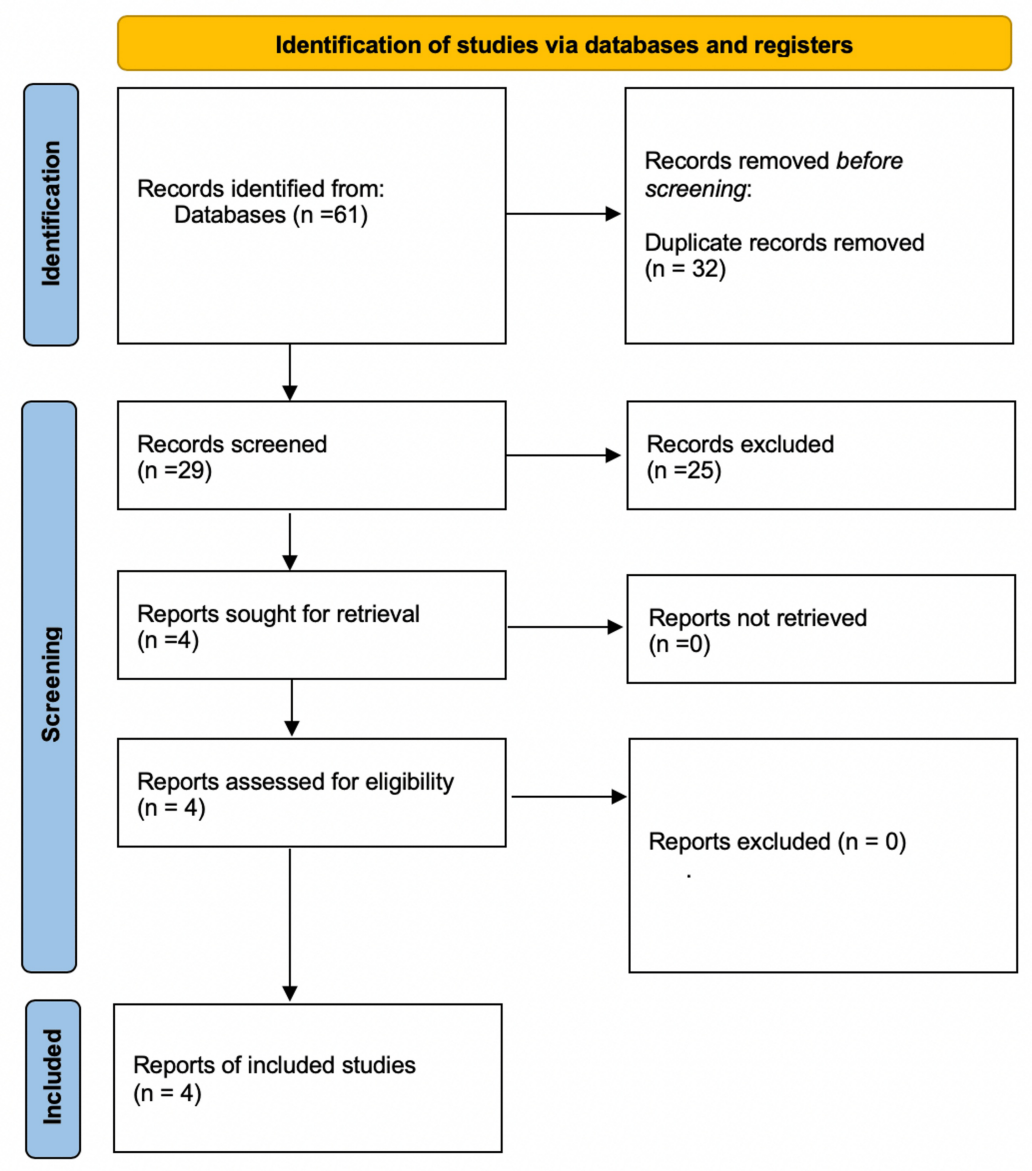
PRISMA flowchart depicting the selection process of the included studies in this review PRISMA: Preferred Reporting Items for Systematic Reviews and Meta-Analyses

Our search in electronic databases such as PubMed, Scopus, and Web of Science resulted in 61 articles. During the selection process, 32 were excluded as duplicates. Thus, 29 studies were screened for inclusion based on the predefined criteria. As shown in the Preferred Reporting Items for Systematic Reviews and Meta-Analyses (PRISMA) flowchart, only four studies were finally included in this review (Figure 1).

This study will focus on the current applications of robotic-assisted surgery in the field of ENT in Greece, the future prospects, and the strategies that other countries have already applied to ameliorate their robotic-assisted ENT surgeries and implement this advanced technology into their everyday surgery programs.

Results

In Greece, robotic surgery in the ENT field is scarcely reported in peer-reviewed studies (Table 1) [4,5,8,9]. Minimally invasive techniques, including robotic surgery, are currently used, but only a few surgeons and clinics share their surgery results, either by comparing these modern techniques to the classic ones or by presenting the advantages and complications of minimally invasive surgeries in ENT.

**Table 1:** Summary of basic characteristics of the included studies F: female; M: male; TORS: transoral robotic surgery

Study	Duration	Participants	Surgery	Results
Kiriakopoulos and Linos, 2012 [4]	2012	8 (6F + 2M) robotic group vs 4F endoscopic group	Robot-assisted thyroidectomy through gasless transaxillary approach vs endoscopic procedures	Both are safe and feasible, with similar results, and an excellent view of the critical neck anatomy allowing precise tissue handling/dissection. Endoscopic approach results in significantly faster and convenient thyroidectomy
Pataridou, 2013 [7]	2010-2013	14	TORS	TORS allows procedures equivalent to traditional transoral surgery but with the advantage of 3D HD visualization of the laryngopharyngeal structures and precision/dexterity by robotic instrumentation
Linos et al., 2013 [8]	January 2000-March 2010	596	Thyroidectomy (open conventional)	Evaluation of patients’ attitudes toward transaxillary robot-assisted thyroidectomy. Only 11.6% of the patients would prefer to have been treated with the transaxillary method. Reasons: 39.2% state that it is more painful, 25.4% are unsatisfied with the longer duration of the robotic technique, 29.1% are satisfied with aesthetic result of open surgery, 15.5% would not choose robotic technique due to the higher cost. Those with bad scarring of the neck after the conventional surgery were more favorable toward robotic method (p = 0.025). Patients with benign/uncertain neoplasm (p = 0.022) or younger patients (p = 0.003) held a more positive view of the new method
Moraitis et al. 2021 [5]	2018-2021	11	TORS	Six therapeutic surgeries with microscopic negative margins resulted in 5 patients free of disease. Five diagnostic surgeries (two primary tumors) had no serious complications

Transoral robotic surgery (TORS) is a rapidly evolving surgical technique that offers minimally invasive surgical practice for head and neck diseases since 2009, when it was widely accepted and FDA-approved [5]. This technique enables precise tumor excision in challenging areas, such as the base of the tongue, while minimizing damage to surrounding tissues. It offers high-quality surgical results with very satisfactory cosmetic outcomes and a much shorter recovery period, while it also reduces the need for adjuvant therapies. Thus, this technique’s advantages make it an attractive and desirable alternative for patients and surgeons [1].

The first successful TORS performed in Greece took place in a large private clinic in Athens, in 2010. The patient was a 52-year-old man who underwent a resection of a solid mass from the epiglottis, located just above the vocal cords. During the operation, a unique navigational system provided only by the da Vinci Si HD system was used [10]. Since then, it has been a surgical technique used primarily for malignancies located in the head and neck region, and it is one of the most frequently performed robotic surgical procedures in ENT in Greece, especially for treating oropharyngeal carcinomas.

Based on the included studies, this technique features very high rates of negative surgical margins among Greek patients. Additionally, no serious complications were reported, and postoperative complications, including dysphagia and aspiration, were low among patients who underwent TORS [5-6,8].

Today, many trained surgeons offer TORS in the private healthcare setting, but unfortunately, very few cohorts or patient data have been published [5,8]. TORS in Greece is performed for benign or malignant tumors, as well as obstructive sleep apnea (OSA), with good results in follow-up while taking advantage of the new robotic systems that offer better results, 3D visualization, and precision due to robotic instrumentation. Unfortunately, this technique is mostly available in private hospitals in the large Greek urban centers (Athens and Thessaloniki) where advanced robotic systems are accessible. Till today, there has yet to be a study referring to TORS performed in a Greek public hospital, mainly due to the unavailability of robotic systems due to budget constraints that have hindered widespread adoption in public hospitals [8]. Only very recently, on May 24, 2024, the first da Vinci robotic system ever installed in a Greek University Hospital, Aretaieio, was inaugurated [11].

The use of robotic surgery as a treatment option for OSA remains minimal in Greece. Only a small number of cases were reported in specialized centers across the country [8]. TORS for OSA involves the removal of obstructive tissues from the tongue base which is a common obstruction site in moderate to severe OSA patients [12-14]. While TORS for OSA has shown promising results in other European countries, like Italy and the UK, there is still insufficient data to establish its efficacy within the Greek population [15-19].

A recent study in a private clinic in Athens found that TORS significantly reduced the apnea-hypopnea index (AHI) in patients who did not respond well enough to CPAP therapy [6]. The study reported an average AHI reduction of 50%. Also, improvements in sleep quality and daytime alertness were reported. However, this procedure is not widely used in Greece due to the procedure's high cost and limited availability of surgical robotic systems. For example, the Da Vinci surgical system costs about $1.2 million, maintenance costs of $100,000 per year, and $1500 worth of disposable surgical instruments are needed per patient or per case [7]. While TORS for OSA shows a lot of potential, more clinical data and expanded access to robotic technology are needed for it to become routine practice in Greece [6,12].

Robotic techniques are also currently used in other types of surgery, such as thyroidectomy and parathyroidectomy. These two surgical procedures are commonly performed, even in young adults, and they may lead to bad cosmetic results if the surgeon ignores the aesthetic surgery principles [20]. Neck incisions may form keloids, and as the neck is an exposed part of the body all year long, the cosmetic result of the incision is of high importance for both men and women. The surgeons may try to perform thyroid surgery from a small incision made in a skin crease of the neck, but an excessively small incision may lead to unpleasant scarring and also a shorter incision leads to a narrower surgical space, which makes it unsuitable for large thyroid volume or nodules [18]. However, patients seem to be concerned about the cosmetic results of robotic thyroidectomy, as the incision is made lower on the neck, thus being more visible than the traditional “open” procedure [9,21,22].

Robotic surgery is emerging as a viable alternative to traditional open surgery in Greece. The majority of patients choosing this technique are very concerned regarding the aesthetic outcome. In comparison to endoscopic thyroidectomy, employing a robotic system in an endoscopic approach through the axilla offers superior visualization of the thyroid bed. The robot's wrist action offers a superior range of motion compared to basic endoscopic tools, while tremor is also eradicated [9,21,22].

Rigorous criteria disqualify patients from robotic thyroidectomy, including previous cervical surgeries, antecedent vocal fold paralysis or a history of voice or laryngeal disorders necessitating treatment, malignancy with extrathyroidal invasion, multiple cervical lymph node metastases, perinodal infiltration at a metastatic lymph node, distant metastasis, and a lesion situated in the dorsal region of the thyroid that may pose a risk of injury to the trachea, esophagus, or recurrent laryngeal nerve during the operation. However, research has shown that robot-assisted thyroidectomy is equally successful and safe as traditional thyroidectomy [22].

A few Greek private hospitals have successfully implemented robotic-assisted thyroidectomy, which avoids sizeable visible neck incisions. Kiriakopoulos and Linos’ study presented eight patients, six females and two males, who underwent robot-assisted thyroidectomy and compared their results to four female patients who underwent endoscopic approach surgery [4]. This robotic-assisted technique is more beneficial for patients with benign thyroid nodules, small thyroid carcinomas, and hyperparathyroidism. However, there is potential that the method may evolve and include a more comprehensive selection of patients in the future [22].

A small study in a private Athenian hospital reported low complication rates in robotic thyroidectomy. Kiriakopoulos and Lino's study presented data from three lobectomies, two total thyroidectomies, two near-total thyroidectomies, and one total thyroidectomy with lateral lymph node dissection. Although the operation time was longer in the robotic group, the complication rates were low. There was only one temporary recurrent laryngeal nerve paralysis in the robotic group, while two patients presented with hypocalcemia [4,9,21,23].

Unfortunately, the procedure's advanced technical demands and the cost of robotic equipment limit its widespread use within the Greek healthcare system. The scarcity of robotic platforms in public hospitals further exacerbates the disparity in access to this advanced surgical option. Operational time is higher, and the cost of the da Vinci robotic system and the instruments needed for the procedure are very high, thus making wide availability impossible either in the private sector or public health system [4,9,24].

A recent study indicates that robotic thyroidectomy in Greece has a success rate exceeding 95%, reduced recovery time, and minimal scarring. These results are similar to those of other European countries, where robotic thyroidectomy has become the gold standard for selected patients [4,5,9].

Discussion

In the Greek private healthcare sector, approximately 30%-40% of ENT surgeries involve some robotic technology, compared to 60%-70% in countries like Italy and Germany. The adoption rate in Greek public hospitals remains below 10%, highlighting the disparity in access to advanced surgical technologies [4-6,8-9,15,17,25-28].

ENT patients in Greece who undergo robotic surgery report outcomes that are similar to those reported by their European counterparts. For example, the five-year survival rate for head and neck cancer patients treated with TORS in Greece mirrors that of Italy and the UK, at around 80%. Furthermore, complication rates for robotic thyroidectomy in Greece are low, consistent with findings from other European studies [4-6,8,17,19,23,25-29]. Table 2 summarizes the comparison, as mentioned above, between Greece and other countries.

**Table 2:** Data in Greece and other European countries in robotic surgery for ENT cases ENT: otolaryngology

Study	Country	Complication rate (%)	Five-year survival rate (%)	Adoption rate
Aubry et al., 2011 [26]	France	Approximately 5%-8%	High short-term recovery rates	Increasing in specialized hospitals, exact rate unspecified
Pataridou A., 2013 [7]	Greece	7.2%	80.5%	Private hospitals: 35%; public: 8%
Makitie et al., 2018 [24]	Denmark	4%-5%	85%	Limited to specialized centers
Makitie et al., 2018 [24]	Sweden	2%-7%	83%-86%	Active in four out of seven major centers
Makitie et al., 2018 [24]	Norway	Approximately 5%	84%-87%	One active center
Makitie et al., 2018 [24]	Finland	4%-6%	Not specified	Active in two out of five university hospitals
Mandapathil and Meyer, 2021 [25]	Germany	2%-6%	Not explicitly detailed	University: 21.4%; non-university: 0.04%
Rao and Gangiti, 2021 [23]	UK	Similar to Italy, not precisely quantified	High, similar to Italy	Increasing in specialized and private centers, specific rates not mentioned

Robotic ENT surgery techniques have been used in Greece for over a decade, but published data are scarce. There are no big cohorts or case series that represent the Greek population in literature and global statistics. This condition underlines the need for high-quality and adequately designed prospective studies for robotic surgery specifically in the ENT field, either TORS or thyroidectomy and parathyroidectomy.

Various reasons play a significant role in this difference in adoption rates between Greece and other European or developed countries. In addition, other specialties like orthopedics, general surgery, and urology have adopted robotic techniques for various surgery procedures. However, access, retention of the surgical plane, and positioning of the robotic system are more accessible for most of these surgeries, compared to the strictly limited space and anatomy of ENT surgical procedures. Due to the need for many adequately trained surgeons capable of using the da Vinci systems, associations and medical teams of the specialties mentioned above have created programs for acquiring skills in robotic surgery [17,30-37].

Greece needs more trained robotic surgeons in ENT than other European countries. A survey conducted in 2022 revealed that only 20% of Greek ENT surgeons have received formal robotic surgery training, compared to 50% in Germany and 60% in Italy. This expertise gap is why the slower adoption rates of robotic surgery in Greece, especially in rural areas where access to training programs and robotic technology is limited [38].

The need for educational programs in Greece poses a challenge to expanding robotic surgery use. Collaboration with international centers, such as those in Italy and the UK, could help bridge this gap by providing Greek otolaryngologists with opportunities for advanced training and certification in robotic surgery. As robotic systems are not widely available even in large public hospitals of the largest cities of Greece, such as Athens and Thessaloniki, residents and young doctors need to travel abroad or seek training in the private sector after finishing their specialty training [17,19,27,38,39].

Inadequate national training programs for robotic surgery have hindered the emergence of experienced surgeons in Greece. Currently, most Greek robotic surgeons have obtained training abroad, primarily in Italy, Germany, and the UK. This practice creates a barrier for young ENT doctors to educate themselves in robotic surgery, as they need to have the budget, ability to travel, and free time to travel abroad or work extra hours so that they gain experience in robotic surgery techniques. Domestic training programs are critical for growing the number of skilled robotic surgeons in Greece and providing widespread access to modern surgical treatment [11,17,19,22,23,36,38].

There are also other barriers that play a significant role in limiting ENT robotic surgery. The economic constraints are a barrier in performing more surgeries with the most advanced robotic methods. The high cost of obtaining and maintaining robotic systems, along with Greece's continuous economic woes, limit the expansion of robotic surgery in public hospitals. A single da Vinci robotic system can cost more than two million euros, making it out of reach for many public healthcare facilities. As a result, the majority of robotic procedures in Greece are conducted in private facilities, with patients often paying out of pocket or using private insurance [38,40,41].

Greece is also a country where most of its population is gathered in the main cities, but there are various smaller hospitals that have a significant role in the healthcare system. These hospitals may have (or not) an ENT department with trained physicians where surgeries are performed. As already mentioned, access to robotic surgery is limited to major cities like Athens and Thessaloniki, resulting in underserved rural populations. This uneven distribution of robotic devices across the country exacerbates healthcare disparities by forcing rural patients to travel long distances for robotic surgery [38,40,41]. Expanding telemedicine and remote robotic surgery capabilities may help to lessen geographic disparities and improve rural patients' access to care. Installing 5G in many geographical regions of Greece may also lead to more safety in rural or distal areas and islands with inadequate health coverage [42-48].

If the authorities make careful decisions in the future, ENT departments may provide high-quality services with the latest technologies and surgical advances. Although ENT doctors are adequately trained for open surgeries and laparoscopic methods, robotic surgery falls behind [38].

Investment in healthcare infrastructure is of very high importance. Government investment and public-private sector partnerships could improve access to robotic surgery in Greek public institutions. Investing in new robotic systems and updating current facilities will allow the Greek healthcare system to provide more egalitarian access to modern surgical care [49].

Training young doctors in robotic surgery is essential, mainly if it is provided during their residency. Thus, all ENT doctors who get their specialty license and have the ability to operate would be capable of operating safely and efficiently, as they would be trained with the latest technologies. Robotic surgery skills should be a prerequisite to getting the ENT specialty license. Establishing national training programs in partnership with foreign centers of excellence is crucial for developing a proficient robotic surgery workforce. Offering hands-on robotic surgery training and certification can assist in expanding the number of skilled surgeons in Greece while improving patient care [11,38,41,42,46]. For example, in the United States, most general surgery resident programs offer a formal robotic surgery curriculum in the first three years of residency, including thyroidectomy procedures [50].

Finally, expanding the regions where surgical technology and other ENT technological advances are offered as treatment options would benefit the Greek population. Increased hospital access to robotic surgery, especially in rural locations, may help close the current gap in patient care. Furthermore, improvements in telemedicine and remote robotic surgery capabilities may be essential to increasing Greece's access to robotic surgery by enabling patients in isolated areas to benefit from the experience of surgeons in larger cities [11,38,43,45-47].

## Conclusions

Robotic surgery in ENT has shown great promise in Greece, particularly in treating head and neck cancers. However, cost constraints, limited access, and a shortage of skilled experts must all be addressed to completely integrate robotic surgery into the Greek healthcare system. By investing in infrastructure training and service extension, Greece can maximize the benefits of robotic surgery, potentially ameliorating patient outcomes and quality of life. This study summarizes the literature search results of ENT robotic surgery studies in Greece and compares the results with other European countries in order to indicate the need for further research and appropriate changes.
